# Do autism spectrum disorder and anorexia nervosa have some eating disturbances in common?

**DOI:** 10.1007/s00787-018-1188-y

**Published:** 2018-07-04

**Authors:** Louise Karjalainen, Maria Råstam, Gunilla Paulson-Karlsson, Elisabet Wentz

**Affiliations:** 1 Gillberg Neuropsychiatry Centre, Institute of Neuroscience and Physiology, University of Gothenburg, Kungsgatan 12, SE-41119 Göteborg, Sweden; 2 Anorexia-Bulimia Unit, The Queen Silvia Children’s University Hospital, Göteborg, Sweden; 3 Child and Adolescent Psychiatry, Department of Clinical Sciences Lund, Lund University, Lund, Sweden; 4 Department of Clinical Neuroscience, Karolinska Institutet, Stockholm, Sweden; 5 Department of Psychiatry and Neurochemistry, Institute of Neuroscience and Physiology, University of Gothenburg, Göteborg, Sweden

**Keywords:** Eating disorders, Eating behaviours, Anorexia nervosa, Autism spectrum disorder, SWEAA, AQ

## Abstract

A possible overlap between autism spectrum disorder (ASD) and anorexia nervosa (AN), in terms of both behavioural and cognitive features, has led to new areas of research. The aim of the present study was to examine the occurrence of eating behaviours frequently seen in ASD among adolescents and young adults with AN. The participants were females within the age range 15–25 years: 36 with current AN (32 were followed up after 1 year), 19 with ASD, and 30 healthy females. The participants completed the SWedish Eating Assessment for Autism spectrum disorders (SWEAA) and the Autism Spectrum Quotient tool (AQ). AN groups had significantly higher SWEAA scores than the healthy comparison group, also when patients had gained weight. Typical autistic eating behaviours, such as selective eating, were more common in the AN groups than in the ASD group. This is the first time that SWEAA has been implemented in an AN population. Eating behaviours frequently seen in ASD seem to be frequent in AN and some remain also after weight gain.

## Introduction

Similarities between autism spectrum disorder (ASD) and anorexia nervosa (AN) were first suggested in the 1980s [1]. Since then it has been reported that autistic conditions may precede AN [2, 3]. A possible overlap between behavioural and cognitive features has led to new areas of research [4–7]. It is well established that certain neuropsychological functions are impaired in individuals with ASD (e.g. cognitive flexibility, such as set-shifting, and central coherence) and that those impairments are often recognised in the clinical presentation of AN [4, 8–14]. Individuals with AN are often socially withdrawn with few friends and obsessive behaviours [5, 15] and present with weak central coherence [11, 16], as well as impaired theory of mind [17].

Selective eating (such as eating a limited number of foods; for instance, only eating food items of a certain colour) and pica (eating inedible things) are examples of typical aberrant eating behaviours in individuals with ASD [18]. In 2013, the SWedish Eating Assessment for Autism spectrum disorders (SWEAA) was designed and validated by our research group, to assess eating behaviours frequently seen in ASD and problems with eating and mealtime surroundings in ASD [19]. The SWEAA has been validated and used to show differences between ASD patients (over 15 years of age, with normal intelligence) and healthy controls [19]. The study showed that ASD patients exhibited specific difficulties with the social situation surrounding food and eating, as well as with simultaneous capacity (doing two things at the same time), rather than with typical eating disorder symptoms.

The Autism Spectrum Quotient (AQ) has recently been used to measure autistic traits in adults with AN [20]. Patients with AN scored significantly higher on the AQ compared with healthy controls [20]. Further studies reported that AN patients exhibited autistic traits, in the range between ASD and healthy controls [21]. Conversely, it is of outmost importance to acknowledge all possibilities, e.g. a consequence of starvation, of elevated scores on ASD measures in AN populations during the ill-state [22, 23]. Furthermore, it has been suggested that autistic traits should also be investigated in recovered AN individuals, to determine whether the autistic behaviour is a state during the AN phase rather than a lifelong trait [21]. Premorbid autistic traits have, in turn, been shown to be a predictor of a poor outcome for teenage onset AN [24]. To our knowledge, specific eating behaviours frequently seen in ASD have not previously been explored in individuals with AN.

The aim of the present study was to examine the occurrence of eating behaviours frequently seen in ASD among adolescents and young adults with AN. As cognitive as well as bodily functions are greatly impaired by low body weight/low BMI, we hypothesised that eating behaviours frequently seen in ASD occur in individuals with AN during the starvation phase and, in most cases, subside with weight gain. We further hypothesised that individuals with AN would exhibit SWEAA scores between those of individuals with ASD and a healthy comparison group, with individuals with ASD having the most deviant results.

## Methods

### Participants

Thirty-six females with current AN (AN-C) (body mass index (BMI) ≤ 17.5 kg/m^2^), all diagnosed by a psychiatrist according to the DSM-IV [25] and fulfilling the DSM-5 [26] AN criteria, were recruited from the Anorexia-Bulimia Unit at the Queen Silvia Children’s University Hospital, Gothenburg, Sweden. All but five were in the age range 18–24 years. Five females were between 15 and 17 years old and two of them (age 15:9 and 15:0) had a BMI < 17.5, but were not underweight by percentile for age and gender, corresponding to the 7th and the 12th‰, respectively. These two adolescents fulfilled all the other criteria for AN according to the DSM-IV and DSM-5 and were classified as Atypical AN (DSM-5). The AN-C group was followed up after 1 year (AN-1 yr), regardless of whether they had recovered or not. Of the original 36 individuals, four could not be reached despite several attempts, leaving 32 individuals to make up the AN-1 yr group. Not all of the AN-1 yr individuals had recovered in terms of weight (13 individuals had a BMI ≤ 18.5), but at group level, the BMI had increased significantly at follow-up (see Table 1). The AN-1 yr group consisted both of individuals still in treatment and individuals who had finished their treatment.

**Table 1:** Demographic information, SWEAA and AQ data for all groups

	AN-C *n* = 36	AN-1 yr *n* = 32^d^	Change AN-C to AN-1 yr	ASD *n* = 19	ASD vs. AN-C *p* value	COMP *n* = 30
Age	19.6** (2.23) 15–24	20.7*** (2.30) 16–26	1.1 (0.07) 15–26	18.5 (3.41) 15–24	*p* = 0.15	18.0 (2.47) 15–23
BMI^a^	16.1*** (0.89) 14–17.5	18.2*** (1.66) 16.2–25.1 (*n* = 31)	2.1 (0.77) 14–25.1 *p* < 0.001	23.2 (5.46) 15.57–36.89	*p* < 0.001	21.3 (2.18) 17.4–26.1 (*n* = 29)
SWEAA_total score_	32.0*** (13.8) 8–56	25.6*** (13.4) 5–53	− 6.4 (− 0.4) 5–56 *p* = 0.009	22.3** (16.0) 3–55	*p* = 0.013	11.0 (3.65) 2–25
SWEAA_BTSD-score_^b^	28.0*** (18.3) 6.25–72.5	25.0*** (17.2) 3.75–63.75	− 2.99 (− 1.03) 3.75–72.5 *p* = 0.67	21.8*** (15.8) 2.5–53.75	*p* = 0.24	8.83 (4.63) 1.25–25
AQ	17.6** (8.2) 5–41 (*n* = 34)	14.13 (7.0) 4–36	− 3.47 (− 1.2) 4–41 *p* = 0.015	N/a	N/a	10.71 (4.71) 7–21 (*n* = 14)
AN duration^c^	2.73 (3.02) 0.25–12	3.80 (2.81) 1.5–13.1	1.07 (− 0.21) 1.25–1.1 *p* < 0.001	N/a	N/a	N/a

Continuous variables shown as mean (SD)/min–max

*SWEAA* The Swedish Eating Assessment for Autism spectrum disorders, *AQ* The Autism Spectrum Quotient, *AN-C* current anorexia nervosa, *AN-1* *yr* anorexia nervosa follow-up after 1 year, *ASD* autism spectrum disorder, *COMP* comparison group

*p < 0.05, ** *p* < 0.01 and *** *p* < 0.001 statistically significant difference compared with COMP

^a^BMI: body mass index, calculated as: weight (kg)/height (m)^2^

^b^BTSD-score: best two subscale discriminating score = mean of the subscales Social situation and Simultaneous capacity

^c^AN duration in years at admittance/start of treatment

^d^Data for the four non-participants in the follow-up shown as means (SD): age: 21.5 (1.73), BMI: 16.7 (0.67), duration: 3.75 (4.9), SWEAA total score: 42 (9.1), SWEAA BTSD-score (i.e. social situation and simultaneous capacity): 45.3 (20.7), AQ: 19.8 (11.6). Although no significant difference, the four abstainers had higher means for illness duration, on both the SWEAA scores and the AQ scores, compared with the non-abstainers

A group of 19 age-matched female patients with ASD (ASD) recruited from a specialist unit, the Child Neuropsychiatric Clinic, Queen Silvia Children’s University Hospital in Gothenburg, was included in the study. All participants were within the age range of 15–25 years at the time of inclusion and had been thoroughly neuropsychiatrically and neuropsychologically evaluated. Their ASD diagnoses were assigned based on DSM-IV criteria, which were the criteria in place at the time of the assessment.

In addition, a comparison group (COMP) of 30 healthy females, group-matched for age, was recruited from high schools and universities in the Gothenburg area through school nurses and advertisements.

The results from the present study were compared with data from the original validation study of the SWEAA [19]. In the original SWEAA study, both females and males were included whereas all the participants in the present study were females. Only the females from the ASD group and the females of the healthy comparison group in the original study were included in the present study comparing SWEAA and AQ data. To determine cut-off scores for the SWEAA, based on the results from the original validation study, both male and female data were used to create a non-gender-specific cut-off.

#### Demographic information

The AN-C group was significantly older than the COMP group. There was no significant difference regarding age between the AN-C and ASD groups. The demographics for all groups can be seen in detail in Table 1. For the four participants with AN who did not participate in the follow-up after 1 year, there were no significant differences in terms of age, BMI, or illness duration at baseline compared with the participants who completed the 1 year follow-up assessment. Means and SD for these four participants regarding age and illness duration can be found in Table 1.

### Procedure

The AN-C group was asked to participate in the study in connection with their regular visit to the clinic, and was invited to participate in a follow-up assessment after 1 year (AN-1 yr).

### Instruments

The SWedish Eating Assessment for Autism spectrum disorders (SWEAA), a validated self-report questionnaire pertaining to general problems with mealtime surroundings and eating in individuals with ASD and normal intelligence, was distributed to all participants [19]. The items of the SWEAA were developed based on a thorough literature review [18] and the experience, in two of the authors, of clinical work as well as research in the fields of eating disorders and ASD. During the development of the SWEAA outmost care was taken to pinpoint specific eating behaviours known from ASD (apart from one subscale pertaining to traditional eating disorder behaviours). Many traditional eating disorder items were omitted during the validation process as they did not discriminate between individuals with ASD and healthy comparison cases. Although the intent was for the items to grasp typical eating behaviours frequently seen in ASD, the validation process revealed items overlapping with traditional eating disorder symptoms (see subscale G below). In Table 2 each subscale of the SWEAA is presented with an example item. The SWEAA is based on 60 items divided into a number of subscales, all targeting different areas of eating behaviour: A. Perception, B. Motor control, C. Purchase of food, D. Eating behaviour, E. Mealtime surroundings, F. Social situation at mealtime, G. Other behaviours associated with disturbed eating (i.e. G1: “I induce vomiting after meals”, G2: “I use diuretics”, G3: “I use diet pills”, G4: “I diet even if other people think I am too thin”, G5: “I fast”, G6: “I am replacing meals with nutritional drinks/powder”, G7: “It is important that one person (the same person) prepares my food”, G8: “I refuse to eat”), H. Hunger/satiety, I. Simultaneous capacity and lastly, J. Pica (see Table 2 for description of subscales and example items). The SWEAA has shown high levels of reliability in terms of Cronbach’s alpha and good test–retest reliability. Furthermore, the instrument has high construct validity and possesses the ability to measure the underlying constructs as well as good scaling properties. Further details can be found in the original validation of the SWEAA [19].

**Table 2:** Description of SWEAA subscales and example items

Subscales	
A. Perception: reflects sensory input related to food, such as smell, taste, texture or sound	I am oversensitive to certain flavours
B. Motor control: assesses different aspects of movement, such as chewing, spilling or table manners	I find it difficult to chew
C. Purchase of food: concerns the control of purchases; for instance, brands or type of groceries	My food must be of a certain brand
D. Eating behaviour: indicates selectivity in eating, such as certain colours, limited repertoire or trying new foods	I only eat a limited menu, maximum of 10 dishes
E. Mealtime surroundings: reflects routines around mealtimes; for example, where to eat or how cutlery is placed	I have certain rituals around mealtimes
F. Social situation at mealtime: assesses the situation in relation to others at mealtime, such as adapting own behaviour to that of others or enjoying company during a meal	I look down at my food most of the time during a meal
G. Other behaviour associated with disturbed eating: questions of traditional eating disorders, such as fasting, purging or dieting	I induce vomiting after meals
H. Hunger/Satiety: measures if the individual can feel when hungry or full	I feel when I am hungry
Single items	
I. Simultaneous capacity: indicates whether the individual finds it hard to do two things simultaneously during a meal	I find it difficult to do two things simultaneously during a meal, e.g. chewing and cutting the food
J. Pica: measures whether the respondent eats inedible things, such as soil or mortar	I eat things that others consider inedible (e.g. mortar or soil)

Two cut-off values were developed; one to measure the overall eating behaviour and one to target those subscales with items that are best discriminating between individuals with ASD and a healthy comparison group. A cut-off value of 12 points for the total SWEAA score was determined through univariable logistic regression analysis. The two subscales, *Social situation at mealtime* [i.e. F1: “I eat together with the one/ones I live with”, F2: “I eat in my bedroom”, F3: “I adapt my behaviour to others who sit around the table (e.g. table manners, conversation)”, F4: “I like company around a meal”, F5: “I talk during the meal”, F6: “I look down at my food most of the time during the meal”, F7: “I say if I think the food is good (when I am invited for a meal)”, F8: “I thank people for the food (when I have been invited for a meal)”, F9: “I eat with knife and fork”, F10: “I leave the table as soon as the food is eaten” (items F1, F3–F5, F7–F9 are reversely scored)] and *Simultaneous capacity* (i.e. I1: “I find it difficult to do two things simultaneously during a meal, e.g. chewing and cutting the food”), have emerged as the subscales best discriminating between individuals with ASD and healthy comparison cases (i.e. best two subscale discriminating score, BTSD-score). The BTSD-score has a cut-off score of ten points (determined through Stepwise logistic regression analysis) (further information on the SWEAA cut-offs can be obtained through contact with the first author).

The AQ is a questionnaire based on the autism spectrum symptomatology [27]. It comprises 50 items based on five different areas with a suggested cut-off of 32 points to identify clinically significant levels of autistic traits [27]. The five domains include Social skills: which assesses choices in social situations; Attention switching: indicates spontaneity and adaptability to changes in routines; Attention to detail: measures the preference and fascination with details and patterns; Communication: concerns social interaction with others, and the ability to manage and enjoy social situations, and Imagination: assesses the ability to pretend and imagine.

### Statistical analyses

For comparison between groups (AN-C, AN-1 yr, ASD, COMP), the Mann–Whitney *U* test was used for continuous variables. To analyse change over time between AN-C and AN-1 yr, the Wilcoxon Signed Rank Test was used. Spearman’s correlation coefficient was used for all correlation analyses (where partial correlations represent having controlled for a certain variable). Non-parametric tests were used as non-normal distribution was assumed for the data. All significance tests were two-sided and conducted at the 5% significance level.

To select variables discriminating between two groups (i.e. AN-C vs. ASD, AN-C vs. COMP), univariable logistic regression analyses were first performed. To find independent variables discriminating between the two groups, the most significant univariable variables (with *p* < 0.0001) were entered in a stepwise forward logistic regression analysis. As a goodness of fit for the model, the area under the ROC curve was calculated.

### Ethics

The study was approved by the Regional Ethical Review Board at the University of Gothenburg, Sweden (GU264-12).

## Results

### Eating behaviours frequently seen in ASD in AN

Table 1 shows eating behaviours frequently seen in ASD, as measured with the SWEAA in individuals with AN during the starvation phase (AN-C) and at follow-up (AN-1 yr). The overall aberrant eating behaviours subsided at the one-year follow-up (as measured by the total score) at group level. There was, however, no significant decrease in the eating behaviours, as measured by the BTSD-score at follow-up, for the individuals in the AN-C group at follow-up (AN-1 yr). Not all individuals in the AN-1 yr group were weight recovered at the time of assessment. Despite this, there was no significant difference, in either SWEAA-score, between the weight recovered individuals (with BMI above 18.5 kg/m^2^ for those > 18 years: or above BMI − 1.5 SD for those under 18 years of age) and the non-weight recovered individuals.

The SWEAA scores for the individuals with AN in relation to those of the individuals with ASD and the COMP group are presented in Table 1. The two AN groups (AN-C and AN-1 yr) had significantly higher SWEAA scores (both total score and BTSD-score) than the COMP group and significantly higher total, but not higher BTSD-score, compared with the ASD group. For the four participants who did not complete the 1-year follow-up, there were no significant differences in terms of the SWEAA total score or the BTSD-score compared with those who completed the follow-up.

### Autistic traits in AN

The total AQ score decreased significantly in the AN-C group from the first to the second assessment (*p* < 0.05) (Table 1). Furthermore, the AN group scored significantly higher on the AQ than the COMP group in the first (*p* < 0.01) but not the second assessment (*p* > 0.05).

Of the individuals in the AN group, 8.8% (*n* = 3), and 3.1% (*n* = 1) scored above the AQ cut-off in the first and second assessment, respectively, as compared with none in the COMP group.

### Implementation of the SWEAA in AN

Table 3 shows the univariable discriminating items with the most significant differences between the AN-C and the COMP groups (*p* values < 0.01). In these cases, the odds ratio (OR) > 1 and the *p* value were significant, which means that there was a higher risk of belonging to the AN-C rather than the COMP group when scoring deviantly on these items. Based on the univariable predictions, stepwise logistic analyses were carried out with all variables, with *p* < 0.001. Significant independent predictive variables of the AN-C vs. the COMP groups were the items “I find it difficult to eat with friends” (Subscale E), “I eat smaller amounts of food than others” (Subscale D), and “I feel when I am hungry (reversed) (Subscale H). For these three items combined in the multivariable analysis, the area under the ROC curve was 0.971 [95% confidence interval (CI) 0.93–1.00] (adjusted odds ratios with 95% CI for each item can be seen in Table 3).

**Table 3:** Items with significant (*p* < 0.01) univariable and multivariable discriminating variables in AN-C vs. COMP groups

Item	Univariable OR (95% CI)	*p* value	Area under ROC curve	Multivariable adjusted OR (95% CI)
A3. I find it difficult to tell what the food tastes like	2.82 (1.32–6.03)	0.0076	0.67	
A6. I find it difficult to eat dishes where several ingredients are mixed, e.g. stews	5.89 (1.87–18.53)	0.0024	0.76	
A7. I am disturbed by the sound of when I chew certain food, e.g. Swedish cracker	2.33 (1.25–4.36)	0.0079	0.68	
A8. I am disturbed by the sounds others make when eating	1.80 (1.19–2.71)	0.0050	0.70	
A9. I am disturbed by other people talking while I am eating	4.43 (1.69–11.62)	0.0024	0.71	
A10. It is important that the food is sorted on the plate	2.97 (1.70–5.19)	0.0001	0.81	
C1. I buy groceries from a special supermarket/business chain	2.21 (1.40–3.48)	0.0006	0.75	
C2. My food must be of a certain brand	3.10 (1.77–5.42)	< 0.0001	0.81	
C3. If I buy food with someone else, I want to check what goods are purchased	3.59 (2.06–6.23)	< 0.0001	0.88	
D1. I prefer certain food depending on the colour of the food	2.46 (1.35–4.47)	0.0032	0.71	
D2. I eat the same food every day	2.46 (1.36–4.46)	0.0031	0.72	
D3. I avoid trying new food/new dishes	3.37 (1.77–6.44)	0.0002	0.79	
D4. I only eat a limited menu, maximum of 10 dishes	3.56 (1.62–7.80)	0.0015	0.77	
D5. I eat smaller amounts of food than others	4.40 (2.24–8.62)	< 0.0001	0.86	4.82* (1.30–17.92)
D6. I drink excessive fluids	2.82 (1.52–5.26)	0.0011	0.73	
E3. I have certain rituals around meal	12.20 (3.34–44.58)	0.0002	0.85	
E4. I get outbursts at the dinner table	5.46 (1.93–15.42)	0.0014	0.72	
E6. I find it difficult to eat at school/workplace/activity centre or similar	4.41 (2.16–8.98)	< 0.0001	0.85	
E7. I find it difficult to eat with relatives	13.65 (3.83–48.67)	< 0.0001	0.91	
E8. I find it difficult to eat with friends	15.14 (4.40–52.05)	< 0.0001	0.92	11.0** (2.04–59.78)
E9. I find it difficult to eat in the café	20.74 (4.19–102.7)	0.0002	0.91	
E10. I find it difficult to eat in a restaurant	22.86 (4.62–113.2)	0.0001	0.92	
E11. I find it difficult to eat when I am abroad	3.78 (2.03–7.06)	< 0.0001	0.84	
F4. I like company around a meal (reversed)	5.32 (2.34–12.11)	< 0.0001	0.83	
F5. I talk during the meal (reversed)	3.02 (1.48–6.14)	0.0023	0.72	
F6. I look down at my food most of the time during the meal	2.86 (1.53–5.37)	0.0010	0.73	
F10. I leave the table as soon as the food is eaten	2.19 (1.25–3.85)	0.0063	0.68	
G4. I diet even if other people think I am too thin	5.63 (2.19–14.50)	0.0003	0.83	
G5. I fast	3.60 (1.34–9.63)	0.0108	0.70	
G8. I refuse to eat	10.23 (2.38–43.90)	0.0018	0.76	
H1. I feel when I am hungry (reversed)	5.82 (2.57–13.16)	< 0.0001	0.86	4.29* (1.17–15.74)
I1. I find it difficult to do two things simultaneously during a meal, e.g. chewing and cutting the food	20.42 (2.64–157.9)	0.0038	0.77	

The AN-C group scored higher than the COMP group on all items

*AN-C* current anorexia nervosa, *COMP* female comparison group

*p < 0.05 and **p < 0.01 statistically significant difference

Univariably derived items, differentiating best between the AN-C and the ASD groups, with *p* values < 0.01 (Table 4), were entered in a stepwise model. Significant independent explaining variables of the AN-C vs. the ASD groups were the items “If I buy food with someone else, I want to check what goods are purchased (Subscale C), and “I diet even if other people think I am too thin” (Subscale G), with an area under the ROC curve of 0.897 (95% CI 0.82–0.98) for the two of them combined in the stepwise analysis (adjusted odds ratios with 95% CI for each item can be seen in Table 4). The derived items indicate that the individuals with AN had higher scores on items that are traditionally associated with AN rather than ASD.

**Table 4:** Items with significant (*p* < 0.01) univariable and multivariable discriminating variables in AN-C vs. ASD groups

Item	Univariable OR (95% CI)	*p* value	Area under ROC curve	Multivariable adjusted OR (95% CI)
C1. I buy groceries from a special supermarket/business chain	2.29 (1.34–3.94)	0.0026	0.76	
C3. If I buy food with someone else, I want to check what goods are purchased	2.72 (1.65–4.50)	< 0.0001	0.85	2.18** (1.28–3.74)
D1. I prefer certain food depending on the colour of the food	2.92 (1.38–6.16)	0.0049	0.75	
D5. I eat smaller amounts of food than others	1.97 (1.22–3.19)	0.0058	0.73	
D6. I drink excessive fluids	2.43 (1.24–4.75)	0.0097	0.73	
E7. I find it difficult to eat with relatives	2.11 (1.24–3.62)	0.0062	0.74	
E9. I find it difficult to eat in the café	2.33 (1.32–4.09)	0.0033	0.76	
G4. I diet even if other people think I am too thin	3.32 (1.61–6.82)	0.0011	0.80	2.55* (1.12–5.80)
H1. I feel when I am hungry (reversed)	2.74 (1.38–5.45)	0.0040	0.75	

The AN-C group scored higher than the ASD group on all items

*AN-C* current anorexia nervosa, *ASD* autism spectrum disorder

*p < 0.05 and **p < 0.01 statistically significant difference

**Fig. 1:**
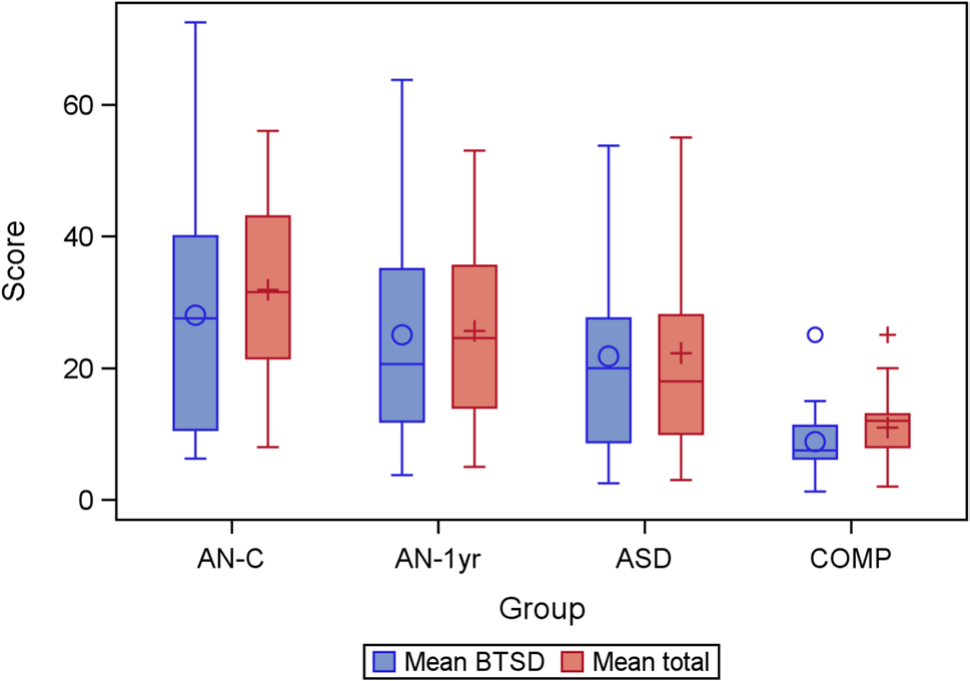
Box plot of mean SWEAA total score and mean BTSD-score for each group. *BTSD-score* best two subscale discriminating score, *AN-C* current anorexia nervosa, *AN-1* *yr* current anorexia nervosa follow-up after 1 year, *ASD* autism spectrum disorder, *COMP* comparison group

The AN-1 yr group did not exhibit higher SWEAA total scores compared with the ASD group (all scores can be seen in Table 1). All groups, except the COMP group, had mean scores above the 12-point cut-off (Fig. 1 shows the mean total scores on SWEAA for all groups).

Based on the mean score from the two subscales distinguishing ASD from healthy comparison cases (F. Social situation at mealtime, and I. Simultaneous capacity), i.e. the BTSD-score, no significant differences were found between the AN-C and the ASD group or the AN-1 yr and the ASD group (all scores can be seen in Table 1). There was no significant difference between the AN-C and AN-1 yr groups. The AN-C group had significantly higher scores than the COMP group, as did the AN-1 yr group. The individuals in the AN-1 yr group did not have a higher BTSD-score than the ASD group. All groups, except the COMP group, had a mean score above the ten-point cut-off (Fig. 1 shows the mean BTSD-scores on the SWEAA for all groups).

For the AN-C group, there was a positive significant partial correlation between the SWEAA mean total score and the AQ score controlled for BMI (Spearman *r*_s_ = 0.58, *p* < 0.001, *n* = 34) (mean AQ scores for all groups can be seen in Table 1). There was also a significant correlation between the BTSD-score and the AQ score controlled for BMI (Spearman *r*_s_ = 0.58, *p* < 0.001, *n* = 34) for the AN-C group.

The AN-1 yr group had a positive significant partial correlation between the SWEAA total score and the AQ score controlled for BMI (Spearman *r*_s_ = 0.61, *p* < 0.001), and a significant partial correlation between the BTSD-score and the AQ score controlled for BMI (Spearman *r*_s_ = 0.51, *p* = 0.004).

No significant correlations were found for BMI and total score or BTSD-score on the SWEAA for any of the groups [AN-C: total score, *r*_s_ = 0.02 (*p* = 0.92), BTSD-score, *r*_s_ = − 0.04 (*p* = 0.81); AN-1 yr: total score, *r*_s_ = − 0.20 (*p* = 0.28), BTSD-score *r*_s_ = 0.04 (*p* = 0.83); ASD: total score, *r*_s_ = − 0.45 (*p* = 0.052), BTSD-score, *r*_s_ = − 0.36 (*p* = 0.14); COMP: total score, *r*_s_ = 0.20 (*p* = 0.30), BTSD-score, *r*_s_ = 0.003 (*p* = 0.99)]. The ASD group had a tendency towards a negative correlation for the mean total score and BMI, i.e. the lower the BMI the higher the mean total score.

## Discussion

To our knowledge, this is the first study to investigate the occurrence of eating behaviours frequently seen in ASD, in young women with AN. We hypothesised that a group of females with current AN (i.e. the AN-C group) would score lower than females with ASD and higher than matched healthy comparison cases on an instrument developed for assessing eating behaviours frequently seen in ASD, the SWEAA. This could not be confirmed in the present study, as the females with current AN had higher total scores on the SWEAA compared with the females with ASD. One explanation could be that eating behaviours frequently seen in ASD are, in fact, more common in AN and also overlap traditional AN eating behaviours. Another possibility is that the patients in the AN-C group, by definition, had a diagnosed eating disorder, as opposed to the patients with ASD, who were not selected based on known aberrant eating behaviours.

In addition, it was hypothesised that eating behaviours frequently seen in ASD could occur in individuals with AN during the starvation phase and, in most cases, subside with weight gain. To some extent this could be confirmed; however, only for the more general eating behaviours (based on the total score). Interestingly, the specific eating behaviours frequently seen in ASD, based on the BTSD-score data, did not subside with weight gain. One can speculate if the eating behaviours frequently seen in ASD have already been present premorbidly to AN and should, therefore, be considered as potential risk factors for the development of the eating disorder. Or, if these behaviours are secondary to the starvation in AN. Furthermore, the behaviours can be transient as well as remaining after recovery. Some evidence implies the presence of autistic traits before the onset of the eating disorder [24, 28, 29]. The autistic traits might also impact the treatment strategies chosen [30]. Alongside this, the eating behaviours frequently seen in ASD can be exacerbated by the starvation of the eating disorder and the social isolation, thus prolonging recovery. However, these issues are not possible to answer based on our findings.

The eating behaviours frequently seen in ASD observed in our group of young women with AN provide important information based on the SWEAA in a population other than ASD, and possibly indicates that total scores may be more inflated by traditional eating disorder behaviours. However, regarding the mean BTSD-score (i.e. based on the subscales Social situation at mealtime and Simultaneous capacity), there was no significant difference between the AN-C and ASD groups. This indicates that eating behaviours characteristic of individuals with ASD also occur in individuals with AN, and overlap ASD aberrant eating. Furthermore, this could support the use of the BTSD-score in addition to the total score, to identify the aberrant eating behaviours frequently seen in ASD.

The AN-1 yr group had a noticeable level of eating behaviours frequently seen in ASD, also among those cases who were weight recovered (as there was no change in mean BTSD-scores at follow-up). The eating behaviours frequently seen in ASD (i.e. the BTSD-scores) appear to persist regardless of weight gain. However, it has to be taken into account that the individuals in our sample were most likely in partial remission at follow-up, which may also have influenced the results.

The most striking differences regarding the SWEAA items between the AN and ASD groups concerned control of purchases and dieting, with the AN group, rather than the ASD group, exhibiting these behaviours. These are ubiquitous symptoms in AN but can also be seen in individuals with ASD, due to rigid and ritualistic behaviours. Specific eating habits, like only eating food of certain textures, is a well-known problem in individuals with ASD and selective eating, but turned out to be more common in the AN group. This could possibly be linked to the obsessive–compulsive and controlling part of the AN symptomatology and could also indicate that these AN traits reinforce the illness, making them very hard to overcome. The ability to feel hunger was significantly reduced in the AN group compared with the ASD group. As expected, regarding traditional eating disorder items, the one with the highest odds ratio for AN was dieting, even though too thin. For AN (compared with COMP), the SWEAA items pertaining to control, dieting and loss of hunger feelings, and problems eating with others, not surprisingly stand out as troublesome.

Females with current AN in this study had a mean score of 17.6 on the AQ, which had decreased significantly at follow-up (AN-1 yr). This is in line with previous studies showing AN patients to have somewhat higher scores than healthy controls, but lower than ASD patients. The AQ scores in the AN-1 yr group were similar to those in healthy comparison groups in previous studies [27]. To our knowledge, no previously published study has measured autistic traits prospectively in a sample of AN individuals. The difficulty of accurately assessing ASD during the starvation phase in AN was illuminated in a recent review of the use of the AQ in individuals with AN [21]. The present study is a first attempt at shedding more light on the presence of autistic traits in AN, before and after weight gain. It appears that some autistic traits, measured with the AQ, are present also after weight gain in some patients, compared with healthy individuals.

Unknown underlying ASD traits in AN may be an aggravating factor in certain psychological treatment modalities [31]. In the acute phase of AN, it is difficult to diagnose ASD accurately [22, 23]. Patients often appear autistic with regard to communication, alongside social withdrawal, rigidity and insistence on sameness. The items in the SWEAA may have different meaning for someone with ASD than for someone with AN, but may however, be equally deviant in both groups. At present, knowledge about a possible overlap between AN and ASD is scarce in clinical settings [32], which leads to a highly unified and standard approach to treatment for all patients. It is of utmost importance for clinicians to be aware of possible autistic traits when patients with AN fail to respond to the standard treatment. The results in this study show that autistic behaviours may be present also after weight gain in individuals with AN and need to be addressed throughout the whole treatment process, also after weight gain. The findings provide an indication of what to search for and explore in AN patients to find specific eating behaviours frequently seen in ASD, including behaviours related to simultaneous capacity and the social situation at mealtimes. Furthermore, this knowledge is important when developing tailored treatment strategies for the group with eating behaviours frequently seen in ASD. Clinical use of the SWEAA in eating disorder units could include identification of patients with eating behaviours frequently seen in ASD in addition to the traditional eating pathology, in order better to understand background factors and tailor the treatment to these patients appropriately from the start. A large study has recently showed, for example, the use of Cognitive Remediation Therapy to be effective for individuals with AN in changing certain cognitive difficulties and thought processes similar to those which can also be seen in ASD [33]. Being able to assess whether there are autistic elements in a patient’s eating behaviour would be valuable to provide adequate individual treatment to each patient. A possible way forward may be looking at what treatment strategies are being developed for the fairly new DSM-5 diagnosis of Avoidant/restrictive food intake disorder (ARFID), and to use pedagogic elements in AN treatment interventions.

## Limitations

The participants in this study consisted of clinical samples. The AN sample was rather small and included only female patients, which affects the generalisability of the results to male samples and to AN in the general population. Originally, the intention was to include males in the AN group, but at the time of recruitment there were no eligible male patients. However, including males in the study would have given additional information on whether eating behaviours frequently seen in ASD are even more common in male patients with AN [34, 35]. Furthermore it is a limitation that the instruments SWEAA and AQ were self-report measurements.

Using the SWEAA in an eating disorder population, pose a potential risk of the total score being high during the acute stage of the disorder, due to the symptoms of the eating disorder rather than any other reason. This is of course a limitation to consider, and it is possible to speculate that for this particular group, or perhaps for all eating disorder types, it would be more valid to use and focus on the BTSD-score rather than the total score. There is also a potential limitation of developing non-gender specific cut-off scores for the SWEAA. Developing gender-specific scores, especially in disorders with unequal gender distribution, would be worth considering for future research and further development of the instrument.

One of the limitations of the present study is that we did not use a specific eating disorder instrument, e.g. the Eating Disorder Examination Questionnaire (EDE-Q) [36] or the Eating Disorder Inventory (EDI) [37] as a means of validating the SWEAA. Both the EDE-Q and the EDI include questions related to food restriction and fasting similar to some of the items found in the SWEAA, the subscale G, pertaining to traditional eating disorder behaviours, in particular. A traditional eating disorder instrument had also been useful to estimate the differences in disturbed eating behaviours between the AN-C and AN-1 yr, and to correlate the data with those of the SWEAA. A next step in future research is, therefore, to validate the SWEAA against the EDE-Q and/or the EDI in individuals with AN. Another limitation is that the discriminant validity of the SWEAA was not assessed, as no similar instrument, evaluating eating behaviours frequently seen in ASD in individuals with normal intelligence and ASD, existed at the time.

There was an age difference between the AN and the COMP group, with the COMP group being slightly younger. However, all the participants, irrespective of group status, were adolescents and young adults within the same age range. Another limitation was the absence of AQ scores for the ASD group and for some of the individuals in the COMP group, why comparisons also had to be made with other AN studies [27].

The last limitation pertains to the scope of this study, which did not include an assessment of the intelligence of the participants. The sample consisted of individuals attending regular compulsory school, which in Sweden is normally synonymous with average intelligence levels but should nevertheless be mentioned as a limitation.

## Conclusions

Eating behaviours known to be typical of ASD have previously not been explored and measured with a validated instrument in individuals with AN. Eating behaviours frequently seen in ASD seem to be far more common in AN than expected and seem to persist regardless of weight gain, whereas autistic traits in general decrease with weight gain.
